# What Is the Meaning of Patient-Centered Decision-Making for a Middle Nurse Manager?—A Qualitative Study

**DOI:** 10.3390/nursrep16010021

**Published:** 2026-01-09

**Authors:** Valeria Di Giuseppe, Raffaella Gualandi, Daniela Tartaglini, Anna De Benedictis, Lucia Filomeno, Daniela Popa, Dhurata Ivziku

**Affiliations:** 1 Department of Bio-Medicine and Prevention, University of Rome Tor Vergata, 00133 Rome, Italy; valeria.digiuseppe@students.uniroma2.eu; 2 Department of Healthcare Professions, San Carlo di Nancy Hospital, 00165 Rome, Italy; r.gualandi@unicampus.it; 3 Department of Nursing, Campus Bio Medico University, 00128 Rome, Italy; a.debenedictis@policlinicocampus.it; 4 Department of Healthcare Professions, Fondazione Policlinico Universitario Campus Bio-Medico, 00128 Rome, Italy; d.popa@policlinicocampus.it (D.P.); d.ivziku@policlinicocampus.it (D.I.); 5 Department of Clinical and Molecular Medicine, Sapienza University of Rome, 00189 Rome, Italy

**Keywords:** patient-centered care, decision-making, health services administration, middle nurse managers (MNMs), nursing, hospitals, qualitative research

## Abstract

**Background**: Patient-centered care (PCC) is a cornerstone of quality, yet its translation into managerial decision-making remains underexplored. Middle nurse managers (MNMs) play a pivotal role in enabling patient-centeredness, but their perspectives on PCC decisions are rarely investigated. **Aim**: This study explored MNMs’ perceptions of what constitutes a patient-centered decision in hospital settings and identified the essential dimensions underpinning such decisions. **Methods**: A qualitative descriptive design was adopted using semi-structured interviews. Thirty-eight MNMs from three hospitals in central Italy were included. Data were analyzed using Elo and Kyngäs’ content analysis approach. **Results**: Two overarching themes emerged as central to patient-centered managerial decision-making (PCMDM): “Meaning and definition of PCMDM,” and “Influencing dimensions of PCMDM”. MNMs described PCMDM as an evolving and adaptable process shaped by patient needs and organizational constraints and unfolding across distinct phases. Key influencing dimensions included the manager’s role, organizational environment, human resource management and knowledge of the patient. **Conclusions**: PCMDM is a continuous, ethical, and reflective process mediated by MNMs, who reconcile institutional priorities, team dynamics, and patient needs to create conditions for high-quality PCC. Implications for Practice: Strengthening PCMDM requires coordinated action aimed at equipping nurse managers with advanced leadership capabilities, building organizational structures that sustain patient-centered decisions, and empowering patients to actively co-shape the care process.

## 1. Introduction

Among the various dimensions that define healthcare quality, patient-centered care (PCC) has emerged not only as a guiding principle but also as a strategic imperative for health systems worldwide. Recognized by international organizations such as the World Health Organization (WHO), PCC is foundational to the delivery of safe, effective, and equitable healthcare [1].

Despite its prominence in global health policies, however, the true meaning of “patient-centered” decision-making for healthcare managers remains insufficiently articulated. More specifically, the ways in which healthcare managers interpret and operationalize PCC in their day-to-day decision-making practices remains a crucial knowledge gap [2]. Without a clear, actionable definition, PCC risks becoming a rhetorical label, frequently invoked but inconsistently applied, thereby undermining the legitimacy and effectiveness of managerial strategies aimed at enhancing patient outcomes. As Bokhour et al. [3] argue, achieving genuine patient-centeredness requires a paradigm shift, one that must permeate all organizational levels, from top leadership down to frontline practitioners.

Healthcare organizations are best understood as complex adaptive systems, characterized by continuous balancing of competing demands, including organizational constraints and patients’ evolving needs [4,5,6]. In these systems, middle nurse managers (MNMs) act as strategic mediators. Positioned at the intersection of top management and frontline professionals, MNMs are called upon to navigate a complex landscape of institutional directives, budgetary constraints, and, most importantly, the diverse, often competing, needs of patients. Research has shown that discrepancies in organizational criteria, patient expectations, and professional interpretations often contribute to fragmentation and inconsistency in the application of PCC, with varying levels of success in aligning these disparate elements [7,8,9]. This fragmentation underscores the critical need for a more systematic approach to PCC that integrates these varying perspectives.

In this context, Osborne et al. [10] conceptualized the public service ecosystem, emphasizing that value creation in public services, including healthcare, cannot be confined to the internal processes of organizations alone. Instead, it is an ongoing, dynamic process involving multiple levels- institutional, organizational, and individual- that coalesce to produce value for patients and society. Their integrative framework emphasizes the creation of value across multiple levels, highlighting the need for public service managers to mediate between societal needs and organizational capabilities [10]. Osborne et al. [11] expands this by introducing a holistic model of value creation, where co-production is just one element of a broader spectrum of processes contributing to value. Though this model is not specific to healthcare, it provides valuable insights into how value can be co-created through collaborative efforts, a concept later applied to healthcare systems by Smith et al. [12]. Smith and colleagues [12] argue that the value generated by healthcare systems should be seen as societal well-being, encompassing health improvement, healthcare responsiveness, financial protection, efficiency, and equity. They argue for a value-based health system, in which managerial decisions must align with broader societal well-being. In this model, patient-centeredness plays a crucial role, but it is framed within a multi-dimensional approach that includes the contribution of all actors in the healthcare system—from policy makers to frontline providers—each contributing to value creation in different ways.

These perspectives converge on a central theme: healthcare decision-making is not merely a technical exercise in resource allocation but a fundamentally human process. Decisions in healthcare must prioritize people, both the patients receiving care and the professionals delivering it, where patient well-being is the primary value. From this standpoint, patient-centered managerial decision-making (PCMDM) rests on three interdependent pillars: first, the recognition and integration of patients’ values, preferences, and needs as guiding criteria for decision-making; second, the alignment of these factors with organizational resources and systemic constraints; and third, the adoption of inclusive, evidence-informed processes capable of creating value for both patients and the broader social ecosystem.

Despite these conceptual contributions, empirical evidence on how healthcare managers interpret and translate patient-centeredness into their decision-making remains limited. Existing research has predominantly focused on the clinical microsystem, enhancing care quality, patient involvement in decision-making, and similar areas [2]. However, the practices of MNMs in supporting and implementing PCC have remained largely underexplored. Studies typically frame PCC either through the lens of patient experience or as part of normative, conceptual frameworks, but little is known about the lived realities of managerial decision-making in hospital settings.

This gap is particularly important because MNMs serve at the critical interface between strategic directives and frontline care delivery. Positioned at this crossroads, they make key decisions that directly shape the organizational conditions under which care is provided. Therefore, understanding how MNMs interpret patient-centeredness and the dimensions that guide their decision-making is essential for advancing both the theory and practice of nursing management. The present study seeks to address this gap by providing an empirically grounded exploration of patient-centered managerial decision-making from the perspective of MNMs. By focusing on their insights, this study aims to offer a clearer and more operational definition of the concept and provide actionable implications that may inform both managerial practice and health policy.

Accordingly, the guiding research question of this study is: “How do MNMs understand and interpret patient-centered decision-making in hospital settings and which dimensions do they consider essential in guiding their managerial decisions?”

### Aims

The aim of this study is to explore MNMs’ perceptions of what constitutes a patient-centered decision in hospital settings and to identify the essential dimensions underpinning such decisions.

## 2. Methods

### 2.1. Design

A qualitative descriptive design was adopted. To ensure rigor and transparency in the methodology, the study adhered to the COnsolidated Criteria for REporting Qualitative Research (COREQ) guidelines (Appendix A).

### 2.2. Setting and Participants

This study involved MNMs who worked in hospital settings and were daily engaged in the management and decision-making processes within their work contexts. Participants were recruited using a purposive sampling strategy, mediated by institutional gatekeepers, to identify MNMs with direct experience in patient-centered decision-making. The research was promoted within the network of the Italian Scientific Society for the Direction and Management of Nursing (SIDMI). Initially, the research was presented to the Directors of Health Professions, and those who showed interest in the study were personally contacted to schedule a meeting, during which further research details were provided and discussed. The Directors identified MNMs within their institutions to be included in the study. Consequently, the researchers contacted the MNMs to explore their availability to participate in the study. After receiving confirmation of their participation, the research team, in agreement with the MNMs, scheduled a suitable time to conduct the interviews.

### 2.3. Inclusion Criteria

Participants were included if they: held the position of MNM or served as interim coordinators for at least one year; had a stable contract with the healthcare organization; were fluent in Italian; had direct experience in decision-making related to PCC; and were actively involved in the operational and decision-making management of nursing teams. These inclusion criteria were essential for providing a comprehensive understanding of the MNM decision-making process.

### 2.4. Exclusion Criteria

Participants were excluded if the MNMs: had recently returned to work after a long absence; had less than six months of experience in the role; or worked in settings that were distant from direct patient care (e.g., MNMs in administrative roles). Those who did not provide informed consent were also excluded. The researchers excluded these MNMs because they lacked a consolidated understanding of the decision-making dynamics and would not have provided relevant input for the study.

### 2.5. Data Collection

Data were collected through individual semi-structured interviews. The interviews were conducted by two researchers with training in qualitative research and experience in nursing and healthcare management. Neither interviewer held a hierarchical or supervisory role in relation to the participants, thereby minimizing potential power imbalances and reducing the risk of response bias. An observer was present during the interviews to note any interruptions or reactions that might influence the discussions.

Interview dates, times, and modalities (online or in-person) were concorded with the MNMs through email. The interviews were conducted during working hours in reserved and quiet rooms, ensuring an environment free from distractions and at a time suitable for both the participants and the research team. Before the interview began, each MNM signed a written informed consent. Interviews lasted between 30 and 50 min and were audio-recorded. Transcriptions were completed within 24 h to ensure accuracy.

At the beginning of each interview, participants completed a brief socio-demographic questionnaire to collect background information (age, gender, tenure as MNM, education, and number of staff guided). Each interview began with the open-ended question “What does patient-centered managerial decision-making mean to you?”, followed by two probes: “In what ways do you think you can translate this process into your daily managerial decisions?” and “What factors most strongly influence your decisions as a middle manager?”. A semi-structured interview guide was used to ensure consistency across interviews while allowing flexibility to explore participants’ experiences in depth. Each interview followed a similar structure, including an introductory phase, the core interview questions, and a closing phase.

Audio recordings and transcripts were securely stored on password-protected devices accessible only to the research team. All identifying information was removed during transcription to ensure confidentiality. Participant enrollment proceeded concurrently with data analysis. Data saturation was considered reached when successive interviews no longer generated new codes, themes, or relevant insights related to patient-centered managerial decision-making. This judgement was informed by an iterative process of data collection and analysis, during which emerging codes and categories were continuously compared across interviews. Once thematic redundancy was observed and no substantial new information emerged, the research team agreed that further data collection was unlikely to add meaningful insights and therefore concluded the interview series. This approach is consistent with recent methodological guidance on documenting data saturation in qualitative research [13].

### 2.6. Data Analysis

Interviews were audio-recorded and transcribed verbatim by members of the research team. Transcripts were checked for accuracy by comparing them with the original audio recordings prior to analysis.

The data were analysed using the method proposed by Elo and Kyngäs [14], which is well-suited for qualitative research focused on extracting and organizing rich thematic insights from interview data. This method is structured into three distinct phases.

In the first phase, open coding, two researchers independently read the transcripts multiple times and made notes within the texts to capture all aspects of the content. Data segments were assigned codes to represent key concepts. In the second phase, category creation, the first-level codes were compared and grouped together to form second-level codes, which were then organized into categories. In the third phase, abstraction, broader themes were developed from similar categories. The categories were further grouped into emerging themes based on recurring patterns. This process helped identify meaningful insights that may not have been immediately obvious. This systematic, three-phase approach was chosen for its clarity and structure in managing and analysing large volumes of qualitative data, ensuring consistency and rigor in the derivation of themes. The approach also facilitated the development of well-defined themes, ensuring that the final interpretation of the data was coherent and grounded in the participants’ experiences.

Two researchers independently encoded and classified the data. All interview transcripts were independently double-coded by the two researchers to enhance analytic rigor. A third researcher reviewed the codes and categories for consistency. Any discrepancies were discussed by the research team and resolved through consensus. Data were organised and analysed using Microsoft Excel (Microsoft Corporation), which was used to support the coding process, the systematic organisation of first- and second-level codes, and the comparison of categories across interviews throughout the analysis.

### 2.7. Trustworthiness

The process of analysis adhered to the trustworthiness criteria proposed by Lincoln and Guba [15], which emphasize rigor, transparency, and reflexivity in qualitative research. To ensure the quality of the findings, four criteria were addressed: credibility, dependability, confirmability, and transferability.

Credibility was established through prolonged engagement with the data, iterative analysis and peer debriefing, and independent verification of codes and categories. Two researchers independently read and coded the transcripts several times, followed by peer debriefing sessions to compare and refine interpretations. Any discrepancies were discussed until consensus was reached, ensuring that the categories faithfully represented the participants’ perspectives and reducing potential researcher bias. Member checking [16,17] was not conducted due to feasibility constraints related to the organizational and managerial context.

Dependability was achieved by maintaining a transparent and systematic process throughout the coding and categorization phases. All methodological decisions were recorded in an audit trail, documenting how categories and themes emerged and evolved over time. This procedure allows other researchers to follow the logical path of the analysis.

Confirmability was strengthened through an independent verification process: a researcher less directly involved in data collection reviewed the first- and second-level codes and the related categories, comparing them with the original transcripts to assess internal consistency and coherence. This strategy minimized subjective influence and ensured that findings were clearly grounded in the data.

Finally, transferability was ensured by providing a detailed description of the study context, the socio-demographic characteristics of participants, and the analytic process. Such a “thick description” enables readers to judge whether the findings can be meaningfully transferred to other settings or populations [18].

### 2.8. Ethical Considerations

This study was carried out in accordance with the ethical standards and principles outlined in the Helsinki Declaration [19] and was approved by the local ethics committee (95.24CET2 cbm, 11 April 2024). The researchers asked for permission from the Board of Directors of each participating centre before the enrolment of MNMs. All participants received adequate information regarding the study and signed the written informed consent form. Data confidentiality was ensured by anonymizing the interviews using a nickname-based coding system. Each participant was assigned a progressive identifier (e.g., *Participant 1*, *Participant 2*, *Participant 3*), corresponding to the chronological order of the interviews. Data access was restricted solely to the research team.

## 3. Results

### 3.1. Sample Characteristics

A purposive sample of 45 middle nurse managers (MNMs) was invited to participate via email. Of these, 38 provided informed consent and were included in the study, whereas 7 did not respond to the invitation. The MNMs worked in three hospitals, two private and one public from the central region of Italy.

Participants had a mean age of 51 (SD 7.3 and ranged 37–62 years). The majority were women (78.9%) with over ten years of managerial practice (52.6%). Most participants (60.5%) had pursued advanced postgraduate training in healthcare management held a Level I Postgraduate Master’s degree. The MNMs worked in different departments across the hospitals. Additional information is present in Table 1.

### 3.2. Findings of the Qualitative Study

The results of the interviews revealed several key themes and categories related to patient-centered decision-making which were grouped under two overarching themes: “*Meaning and definition of Patient-Centered Managerial Decision-Making*” and “*Influencing dimensions Patient-Centered Managerial Decision-Making*” (see Table 2). Below is a description of the major themes, categories, and the frequency of codes that emerged.

#### 3.2.1. Overarching Theme 1: Meaning and Definition of Patient-Centered Managerial Decision-Making

Two themes characterized MNMs narrations of the meaning of Patient-Centered Managerial Decision-Making (PCMDM): “*PCMDM is a dynamic and multidimensional process*” and “*PCMDM unfolds through distinct phases*”.

##### PCMDM Is a Dynamic and Multidimensional Process

Patient-centered managerial decision-making emerged as a dynamic and multidimensional process, consistently described as reflective, rational and evolving, rather than a single act. As one middle manager observed, “*My decision-making [...] during the years has changed thanks to my experience*” (Participant 26, MNM, female, 6–10 years of experience), highlighting the role of professional growth in shaping managerial choices. The process was further characterized as collaborative and multidisciplinary, drawing upon the collective expertise of healthcare teams. One participant emphasized the importance of shared responsibility: “*The challenge for me was deciding together with the nurses how to respond to that indirect health need linked to a bureaucratic and technological barrier, and not to leave a health need unmet*” (Participant 5, MNM, male, 1–5 years of experience).

##### PCMDM Unfolds Through Distinct Phases

Interviews also revealed that managers conceptualized decision-making as unfolding through distinct phases: beginning with data collection and problem analysis, followed by an evaluation of risks and benefits, and culminating in the final decision. The emphasis on weighing potential gains and losses was recurrent: “*The analysis must always be done between losses and gains*” (Participant 26, MNM, female, 6–10 years of experience); “*After reflecting for a long time, after always putting on the scale what is right and what is wrong, you make a decision*” (Participant 14, MNM, female, >10 years of experience).

In sum, decision-making was portrayed not as an isolated managerial act but as a structured, reflective, and team-oriented process, shaped by experience, guided by ethical reasoning, and grounded in systematic evaluation.

#### 3.2.2. Influencing Dimensions Patient-Centered Managerial Decision-Making

Four themes emerged as influencing factors of Patient-centered Decision-Making: “*Role of the managers*”, “*Organizational environment*”, “*Human resources management*”, and “*Knowledge of the patient*”.

##### Manager’s Role

MNMs emerged as the primary actors in PCMDM, positioned at the intersection of organizational responsibility and clinical practice. They were not only seen as coordinators of workflows, but as leaders whose decisions carried direct implications for patient safety, care quality, and staff development.

Their role was described as multifaceted: managers were expected to motivate staff to place the person at the centre of care, to assume responsibility as tutors and trainers for their nursing staff, and to actively participate in the care process by closely observing the dynamics of care delivery, supporting nurses in clinical and organizational decision-making, and intervening in critical moments to maintain the continuity and coherence of patient-centered care. PCMDM was therefore not conceived as a detached managerial task, but as a form of leadership embedded in everyday practice. Motivating staff was described as essential to sustaining a shared vision of care. As one MNM explained, “*Helping the nursing staff to understand that everything we do is meant to support a person going through one of the most difficult moments of their life is fundamental. It is not about making our own work easier or finding shortcuts, but about facilitating the life of someone facing a difficult path. Motivating staff is therefore crucial*” (Participant 11, MNM, female, >10 years of experience).

Active participation in care was also framed as a tangible demonstration of leadership. Another MNM recounted: “*I am the first to step in when there is a shortage of staff or a lack of motivation. You get your “hands dirty” with the nurses, to convey the message that even if we (nursing team) are few, some things must still be done. We may be short in numbers, but the dignity of the patient always comes first*” (Participant 24, MNM, female, 6–10 years of experience). Through these accounts, managers presented themselves as role models, showing that patient-centered decisions are not only made at a desk but also enacted at the bedside, where leadership, teamwork, and patient dignity intersect.

When describing how they made decisions, managers consistently referred to their knowledge, professional experience, and personal values as guiding forces: “*Having scientific knowledge of that area really helps you, as a coordinator, to make decisions*” (Participant 20, MNM, male, <1 year of experience) or also “*Having training in the areas where the patient is admitted is one of the factors that can help focus decision-making on the patient. Many decisions are made about patients, but they are often non-specific. What truly makes a difference is having a deep knowledge of the area*” (Participant 16, MNM, female, 1–5 years of experience). As one manager succinctly stated, “*First of all, ethics*” (Participant 19, MNM, female, >10 years of experience). These elements shaped the lens through which situations were interpreted and options evaluated. Experience provided perspective, knowledge offered tools for analysis, and values ensured alignment with ethical and patient-centered principles.

In this sense, PCMDM was portrayed as a synthesis of competence, reflection, and responsibility. Managers did not simply execute organizational directives; rather, they translated their expertise and values into concrete choices that motivated their teams and safeguarded patients.

##### Organizational Environment

The organizational environment was consistently described as both an enabler and a constraint for PCMDM. Hospital procedures, organizational policies, and institutional hierarchies framed the scope of action, shaping how managers balanced patient needs with organizational demands.

Communication emerged as a key dimension. Managers highlighted the importance of dialogue with peers, noting that decisions were often strengthened through consultation with other MNMs: “*I also take time to evaluate which is the best path; sometimes you consult with other coordinators, who may be more prepared than you because they have already faced the same situation*” (Participant 34, MNM, female, 1–5 years of experience). At the same time, maintaining an open channel with top management was described as essential for aligning patient-centered decisions with institutional expectations. As one participant explained, “*Even those above me in top management recognized this exception to the rule—allowing nurses to take on additional tasks to meet patients’ unmet needs—as an action consistent with patient-centered care, and it was appreciated by the management itself*” (Participant 5, MNM, male, 1–5 years of experience).

MNMs also emphasized the tension between organizational interests, patient needs, and available resources, framing patient-centered decision-making as a continuous act of negotiation. One MNM captured this daily struggle: “*Knowing the patients’ needs in relation to the resources we, as nurse managers, have available is a daily conflict. In an organizational unit, the optimum would be to have an adequate number of staff with specific skills and sufficient material resources. Yet, we must also deal with the company’s demands, tighten the rope, impose restrictions, and pay attention to consumption. What we can—and constantly must—do is reorganize our nursing teams every day, because the goal is always the same: to meet patients’ needs*” (Participant 25, MNM, female, >10 years of experience).

Hospital procedures and policies further influenced decision-making. MNMs acknowledged that institutional directives often set the boundaries within which they operated. One participant reflected: “*Periodically, we, as nurse managers, all have situations imposed from above in our work activities, and we must be the first to respect them and to ensure that they are respected*” (Participant 31, MNM, female, >10 years of experience). Organizational tools and policies were therefore not only regulatory frameworks but also instruments guiding practice, anchoring individual choices to the broader institutional system.

In this sense, the institutional environment was perceived as a structuring force: it provided the procedures and communication channels that sustained patient-centered decisions, but also imposed boundaries that managers had to navigate with flexibility and professional judgment.

##### Human Resources Management

From the perspective of MNMs, the team represented the operational core of PCMDM. Far from being a neutral workforce, it was described as a strategic resource requiring constant alignment between individual competencies, organizational demands, and patient priorities.

A first dimension concerned the assignment of patients according to staff skills. MNMs emphasized the importance of tailoring patient allocations to the nurses’ professional competencies, ensuring that each patient was entrusted to the nurse best suited to their specific needs: “*I assign each patient to a nurse based on their skills. By deciding who will be their nurse, you already understand what the patient needs are, because every nurse has specific skills, and you discuss this with the team*” (Participant 1, MNM, female, >10 years of experience*).* Assigning patients to nurses based on the competencies was thus framed not merely as an organizational duty, but as a managerial responsibility that safeguarded both safety and personalization of care.

A second category centered on balancing group well-being with patient needs. PCMDM was depicted as an act of mediation that protected organizational harmony while ensuring care delivery. As one manager explained, “*If you don’t create excessive stress for your staff, they will most likely be able to provide better care for the person who comes here*” (Participant 33, MNM, male, >10 years of experience). Striking this balance was considered vital for sustaining motivation, preventing burnout, and reinforcing the reciprocal link between staff well-being and quality of care.

Finally, managers underscored that patient-centred decision-making was inherently linked to the pursuit of high-quality care. Ensuring patient and staff safety, guaranteeing appropriate and timely care delivery, and creating optimal working conditions for the team were described as essential components of this process. As one participant explained, “*First and foremost, improving staff well-being, ensuring that they work in the best possible way with all the available resources so that they can deliver care effectively, while at the same time ensuring that patients receive high-quality care*” (Participant 32, MNM, female, 6–10 years of experience). Similarly, another manager emphasised the importance of protecting staff from overload: “*I always try to find the best way for them to work properly, without risks, without overburdening them, so I almost always try to avoid double shifts, only if I am forced to*” (Participant 37*,* MNM, male, 1–5 years of experience). As highlighted by another participant, even workforce planning was oriented towards safety: “*All the decisions a coordinator makes, even when planning the number of nurses and support staff, are aimed at patient safety*” (Participant 9, MNM, female, 1–5 years of experience). To achieve this, MMNs emphasized the importance of developing tailored work plans and adopting strategies planned in advance, pointing to the need for foresight and organizational discipline in team management.

In this perspective, the coordinated team was not simply the setting where decisions were carried out, but a *managerial lever*. Its effective governance allowed MMNs to translate the abstract principles of patient-centeredness into concrete practices of safety, personalization, and organizational well-being.

##### Knowledge of the Patient

The patient remained the core reference point of all managerial choices, confirming their dual role as both recipient and driver of decision-making. MNMs emphasized that patient-centeredness was not an abstract principle, but a daily practice grounded in knowledge of patients and their needs.

A first dimension highlighted the centrality of the patient as a guide to the decision-making process. Patient-centered decisions were framed as acts of responsibility aimed at pursuing a clear goal—working for the patient’s best interest, even when this required making difficult choices. As one manager observed, “*The decision-making is a process with considerable responsibility, because we, as nurse managers, decide for others and must always keep the patient in mind, as the patient is our only objective*” (Participant 4, MNM, female, 1–5 years of experience). Another added, “*Decision-making is fundamental, because many times you must also make uncomfortable decisions that are nevertheless useful for the patient*” (Participant 3, MNM, female, 1–5 years of experience). These accounts illustrate how managers perceived decision-making as a moral duty intertwined with professional judgment.

A second category underscored the need for comprehensive knowledge of patients when making managerial choices. Patient-centered decisions were not only about addressing the needs of a single individual, but also about maintaining awareness of the entire ward population. One participant explained: “*A patient-centered decision also depends on the type of decision. If it is purely organizational, it is not only about one patient, but about all the patients involved. For example, transferring a patient from one bed to another depends on the type of patient next to them and on how you compose the rooms. You must think about the overall patient mix, not only about a single case*” (Participant 22, MNM, female, >10 years of experience).

In this sense, “knowing the patient” extended beyond individual needs to include an understanding of the broader clinical and organizational context, where every decision could affect multiple patients simultaneously. For managers, patient knowledge thus represented both a practical tool for tailoring care and a strategic perspective for orchestrating resources at the ward level.

## 4. Discussion

This study aimed to explore MNMs’ perceptions of what constitutes a patient-centered decision in hospital settings and to identify the essential dimensions underpinning such decisions. The findings confirm that, within the Italian hospital context, MNMs are uniquely positioned to interpret policies, mediate institutional pressures, and enable PCC at the frontline, thus supporting healthcare institutions’ commitment to PCC.

Within contemporary Italian hospitals, MNMs often operate not only under chronic nursing understaffing and sustained workload pressure, but also within broader constraints related to resource scarcity (e.g., limited time, beds, equipment and support services) and organizational rigidities (e.g., budgetary ceilings, procedural requirements and externally imposed targets). These conditions emerged repeatedly in managers’ accounts of daily staffing and care re-organisation, shaping PCMDM as a continuous process of prioritization and ethical trade-offs.

Importantly, despite these structural limitations, MNMs consistently described the patient as the guiding reference point of their decisions: patient-centeredness was portrayed less as an abstract ideal and more as a deliberate effort to preserve dignity, safety, equity, and continuity of care under scarcity—often through harm-minimisation choices and by safeguarding both care quality and the reliability of documentation.

Several MNMs also linked decision-making to workforce instability and contractual arrangements (e.g., temporary contracts, turnover and variable skill mix), which can modify how decisions are enacted in practice—shaping responsibility distribution, team cohesion, and the feasibility of investing in coaching, shared routines, and sustained patient involvement. Under these conditions, decisions were frequently narrated as time-sensitive negotiations between what should be done for the patient and what can be delivered within constraints of staffing, resources, and organizational rules. In this sense, MNMs portrayed PCMDM as a form of buffering work: absorbing system pressures and translating them into actionable, ethically defensible choices that keep the patient’s well-being at the center.

The results suggest that PCMDM is best understood as a dynamic, continuous, and multidimensional process, guided by managers’ professional knowledge, accumulated experience, and personal values, while simultaneously shaped by institutional and organizational frameworks. Based on the results of this study, the PCMDM can be defined as: “*a dynamic, continuous, and reflective process through which nurse managers align organizational constraints, team functioning, and institutional priorities with the overarching goal of safeguarding patient well-being. It is characterized by collaboration, multidisciplinarity, and ethical responsibility, and is enacted not through direct care but by enabling teams to deliver safe, personalized, and high-quality care*.”.

A key finding of this study is the pivotal role played by MNMs, who operate “*at the centre of the stage*” in hospital settings. Positioned between top management and frontline professionals, they act as essential bridges linking institutional directives with clinical practice. Although they are not directly involved in patient care, this study shows that MNMs can significantly influence patient outcomes by creating the conditions necessary for teams to function effectively. In particular, ensuring adequate resources, promoting staff well-being, and fostering organizational cohesion emerged as critical strategies to sustain patient-centered care (PCC). These findings are aligned with previous research. Filomeno et al. [20] emphasize the importance of clearly defining, assessing, and developing managerial competencies as a foundation for effective leadership and for improving both patient and staff outcomes. Gillespie et al. [9], on the other hand, underline the need to establish a shared and multidimensional understanding of PCC among key stakeholders, including managers, to better inform practice and policy.

Furthermore, this study reinforces the idea that PCMDM involves more than simply managing organizational constraints; it also requires personal development and self-reflection. As highlighted by Lommi [21] and Pursio et al. [22], relational, participative, and transformational leadership styles are considered essential for fostering supportive work environments and enhancing nurse autonomy and empowerment. Although autonomy and empowerment did not explicitly emerge from our findings, the interviews revealed that the PCMDM involves motivating staff to provide patient-centered care, taking responsibility as a tutor and trainer, and actively participating in the care process. These aspects reflect a participatory and communicative leadership approach, which the literature associates with increased job satisfaction and stronger commitment to patient-centered care [22,23,24].

Finally, our findings show that MNMs face constant challenges in balancing organizational priorities with patient needs, often requiring them to make quick, informed decisions under pressure. While competencies such as emotional intelligence, conflict resolution, and stress management did not explicitly emerge from the interviews, previous research suggests that these skills are crucial for MNMs to effectively engage their teams and support collaborative and patient-centered decision-making [20]. Together, these findings contribute to a deeper understanding of how MNMs shape patient-centered care through both organizational and relational dimensions, highlighting the need for targeted strategies to develop leadership and emotional intelligence competencies that align managerial practice with patient-centered values.

This study underscores the need for healthcare organizations to prioritize the ongoing professional development of MNMs, providing them with the necessary tools and support to make effective decisions. MNMs, as leaders, coaches, and counsellors, guide their teams through challenging situations, ensuring that patient well-being remains the core focus [22,25,26]. Leadership training programs designed to enhance decision-making skills, along with fostering a collective leadership vision, can empower nurse managers to address organizational challenges more effectively, improving both the quality of care and the work environment.

However, MNMs operate within a complex web of institutional constraints, including limited resources, strict procedures, and regulatory requirements. These findings echo those of Leidner et al. [27], who identified external regulations and the rigid separation of financing and service delivery as barriers to patient-centered care implementation. Despite these challenges, both this study and Leidner et al. [27] suggest that collaboration and information exchange remain pivotal facilitators, ensuring continuity of care and alignment across organizational boundaries. For instance, MNMs in this study emphasized the importance of engaging multidisciplinary teams and fostering effective communication channels to ensure patient-centered care, even within rigid organizational structures. In light of these findings, it is evident that MNMs must develop critical competencies to handle the internal and external tensions inherent in their roles. Conflict management-especially in balancing organizational interests such as cost control with patient needs-emerges as a key competency.

The results of this study indicate that MNMs often encounter moral and ethical dilemmas when making decisions that impact patient care, such as resource allocation in an overburdened system. In these situations, managers must weigh the pros and cons of various options, making informed decisions that are both efficient and patient-centered. Moreover, the literature shows as the emotional intelligence is essential in navigating these tensions. MNMs with higher levels of emotional intelligence are better able to maintain composure, resolve conflicts, and foster positive relationships within their teams, acting as mediators between the organizational system and frontline staff and improve outcomes for both staff and patients [28,29]. To support MNMs in developing all these competencies, healthcare organizations must implement tailored leadership development programs that focus not only on clinical management but also on emotional resilience, ethical decision-making, conflict resolution, and workload management. These interventions will help MNMs balance the workload and foster a patient-centered culture within their teams, ultimately improving both staff well-being and patient outcomes.

As this is a qualitative study, the aim was not to achieve statistical generalisability, but to provide an in-depth and contextually grounded understanding of PCMDM. The strength of the findings lies in their validity and credibility, supported by methodological rigor and analytic transparency. Particular attention was therefore paid to describing the study context, which involved MNMs working in hospital settings within the Italian healthcare system. Importantly, the prominence of prioritization, ethical responsibility, documentation, and continuous re-organisation in participants’ narratives reflects the lived conditions under which Italian MNMs operate; thus, context should be considered not merely a background factor but a core explanatory lens for interpreting how PCMDM is conceptualized and enacted. This contextual grounding supports the transferability of the findings, enabling readers to assess their relevance to similar organizational and healthcare contexts facing comparable resource and workforce pressures.

### Implications for Practice

This study highlights the need for a comprehensive, multi-level strategy to strengthen PCMDM, addressing the roles of managers, healthcare organizations, and patients themselves. Moving beyond individual competencies or isolated interventions, future efforts must aim to create ecosystems that enable managers to lead effectively, institutions to support decision-making processes, and patients to become active partners in care.

MNMs stand at the crossroads of clinical practice and organizational strategy, and their leadership capacity is pivotal to advancing patient-centered care. Healthcare organizations should invest in targeted leadership development programs that go beyond traditional management training to include communication, motivation, coaching, and team-building skills. Scenario-based and simulation training can enhance managers’ ability to make informed decisions in complex and rapidly evolving care contexts, while also equipping them to recognise, nurture, and mobilise the potential of their teams [30,31,32]. By strengthening these competencies, NMs can transform everyday challenges into opportunities to drive innovation and sustain patient-centered practice.

PCMDM cannot rely solely on individual leadership; it requires structural support at the institutional level. Healthcare organizations must create environments that actively facilitate collaboration, continuity, and responsiveness. This includes establishing cross-departmental task forces, fostering regular interdisciplinary dialogue, and integrating decision-support systems to better align organizational strategies with patient needs. Developing policies and governance mechanisms that bridge administrative priorities and clinical realities will help embed patient-centered values into strategic decisions and promote alignment across services and care settings [3,33,34].

True patient-centeredness requires that patients move from being passive recipients of care to active contributors in decision-making. Strengthening communication pathways and engagement strategies is essential to ensure that patient needs, values, and preferences are clearly articulated and systematically integrated into managerial decisions [35]. Approaches such as shared decision-making models, structured communication frameworks, and co-design workshops can foster collaborative relationships between patients and professionals. This not only enhances the quality of decisions but also builds trust, improves adherence, and enriches the overall care experience.

In sum, advancing PCMDM demands coordinated action across all three levels: equipping managers with advanced leadership capabilities, building organizational structures that sustain patient-centered decisions and empowering patients to co-shape the care process. Together, these strategies can foster a managerial culture that is both responsive to organizational contexts and deeply rooted in patient needs, ultimately improving care quality, staff well-being, and health outcomes.

## 5. Limitations and Future Research

This study presents some limitations that must be considered when interpreting the findings. First, as with most qualitative research, the transferability of results is inherently constrained by the specific sample and context. The reliance on a purposive, convenience sample narrows the scope of generalizability, as findings may reflect contextual particularities of this healthcare setting rather than universal patterns. Additionally, participation may have been biased toward those MNMs most attuned or sensitized to the principles of patient-centeredness, potentially limiting the diversity of perspectives captured. Lastly, the study exclusively captured the voices of MNMs, without incorporating the perspectives of middle managers from other professions or top-level executives, frontline nurses, or patients. This professional homogeneity, while analytically useful, restricts a more holistic understanding of PCMDM across different stakeholder groups. However, this study demonstrates several strengths. First, it contributes to the literature by clarifying a concept that has been inadequately defined, offering an initial, precise definition. Second, by interviewing MNMs from both private and public institutions, this study provides a more comprehensive perspective on healthcare and patient care, facilitating a comparative analysis between the two systems and enhancing the clarity of the concept.

Future research should pursue more comprehensive and comparative designs. A priority is the systematic exploration of barriers and facilitators to the enactment of PCMDM, to clarify the organizational and contextual determinants that either enable or constrain its implementation. Second, there is a need for the development and validation of standardized instruments or scales capable of measuring the degree to which managerial decisions at MNM level are patient-centered. Such tools would allow for quantitative assessment and benchmarking across institutions, thereby complementing qualitative insights with broader empirical evidence. Third, comparative investigations across different national and cultural contexts are warranted to explore how managerial decision-making is shaped by diverse health systems, governance structures, and professional cultures. Such research would contribute to building a context-sensitive yet standardized conceptualization of PCMDM, bridging local practices with international policy agendas.

In addition, future research could focus on the development of a comprehensive and operational framework to guide managerial decision-making with a specific focus on patient-centeredness. Such a framework could integrate patient perspectives alongside those of middle managers from different health professions, top-level executives, and frontline nurses, and systematically map the factors influencing managerial decisions. This approach may help distinguish patient-centered from provider-centered decision-making orientations and support managers in aligning organizational processes with patient needs and values.

## 6. Conclusions

This study advances the conceptual understanding of PCMDM by exploring how MNMs define and enact it in everyday practice. Findings show that PCMDM is not a single decision point but a complex, continuous, and ethically grounded process through which managers reconcile institutional directives, team dynamics, and patient needs to safeguard well-being. Its core elements -continuity, adaptability, collaboration, and ethical reflection- are enacted not through direct care but by enabling teams to deliver safe, personalised, and high-quality care.

Patient-centeredness emerges not as a fixed outcome but as an evolving practice of negotiation and reflection that integrates managerial expertise with patient values. Critically, nurse managers achieve this through the team: by motivating staff, sustaining transparent communication, and fostering organisational well-being, they transform patient-centered principles into tangible outcomes of quality, safety, and personalisation.

These findings underscore the strategic importance of team governance as a powerful managerial lever and call for leadership development initiatives that equip nurse managers to embed patient-centered values into decision-making processes. Strengthening these competencies will be key to shaping future healthcare organisations capable of responding to complex system demands while remaining deeply anchored in patient needs.

Future studies should explore the organisational, cultural, and contextual factors that facilitate or hinder the implementation of patient-centered managerial decision-making across healthcare settings. Particular attention should be paid to how communication structures, leadership support, and patient involvement mechanisms act as barriers or enablers of this process. Understanding these dynamics will be essential to design targeted interventions and policies that strengthen the integration of patient-centered values into managerial decision-making and improve outcomes for patients, staff, and organisations.

## Figures and Tables

**Table 1:** Socio-demographic characteristics of the sample (n = 38).

Variables	N (%)
**Age** (mean; SD)	50.13; 7.3
**Sex**	
Female	30 (78.9)
Male	8 (21.1)
**Professional experience**	
1 Year	2 (5.3)
1–5 Years	10 (26.3)
6–10 Years	6 (15.8)
>10 Years	20 (52.6)
**Education qualification**	
Postgraduate Master’s degree (Level I)	23 (60.5)
Master’s of Science Degree (Second Cycle)	10 (26.4)
Postgraduate Master’s of Science Degree (Level II)	3 (7.9)
PhD	2 (5.2)
**Healthcare organization typology**	
Public	8 (21.1)
Private	30 (78.9)
**Number of people managed**	
<20 people	8 (21.1)
>20 people	30 (78.9)

**Table 2:** Overarching themes, themes, categories, codes, and frequencies describing patient-centred managerial decision-making (PCMDM).

Overarching Theme	Theme	Category Major	Code	Frequency
Meaning and Definition of Patient-Centered Managerial Decision-Making (PCMDM)	PCMDM is a dynamic and multidimensional process	PCMDM is a continuous and iterative process.	PCMDM is continuous	3
			PCMDM is rational and evolving	4
			PCMDM is reflective	5
		PCMDM is collaborative and multidisciplinary	PCMDM is collaborative	13
			PCMDM is multidisciplinary	10
	PCMDM unfolds through distinct phases	PCMDM is an analytical process	PCMDM initiates through data collection and problem analysis	3
			PCMDM is based on daily evaluations	2
		PCMDM is informed by risk–benefit assessment and patient context	PCMDM is based on evaluation of risks and benefits	3
			PCMDM includes a series of decision-making tailored to patient situations	1
Influencing dimensions PCMDM	Manager’s role	The coordinator	Motivating staff to care for the person	3
			Taking on the responsibility of being a tutor/trainer for the staff	4
			Actively participating in the care process	8
		Knowledge, experience, and values influence the decision-making process	Basing on experience	9
			Basing on knowledge	14
			Acting according to one’s own values	4
	Organizational environment	Hospital communication	Good communication among coordinating colleagues	4
			Balancing the interests of the organization with the patient’s health needs and the available human resources	4
			Communicating with top management	4
		Hospital procedures influence managerial decisions	Being guided by organizational tools	2
			Sharing organizational policies	5
	Human resources management	Assigning patients based on skills	Knowing the skills and competencies of colleagues is necessary to assist the patient	4
			Managing care by assigning patients based on nurses’ skills	4
		Balancing the group and the patient	Creating organizational well-being	3
			Balancing between the team management and needs’ patients	20
		Ensuring quality care	Ensuring the safety of both the patient and the healthcare professional	4
			Ensuring appropriate care	5
			Enabling staff to work at their best	12
			Developing and organizing tailored work plans	11
			Having a work strategy (planning in advance)	4
	Knowledge of the patient	The centrality of the patient as a guide to the decision-making process	Working for the patient and having a clear goal	7
			Working for the patient, even when it requires making difficult choices	2
		Knowing the patient to make managerial decisions	Knowing the patient and their needs	9
			Knowing all the patients in the ward	2

## Data Availability

The data presented in this study are available on request from the corresponding author due to ethical restrictions.

## Public Involvement Statement

No public involvement in any aspect of this research.

## Guidelines and Standards Statement

This manuscript was drafted in accordance with the Consolidated Criteria for Reporting Qualitative Research (COREQ) guidelines for qualitative research.

## Appendix A

**Table A1:** Consolidated criteria for reporting qualitative studies (COREQ): 32-item checklist.

No. Item	Guide Questions/Description	Reported on Page
**Domain 1: Research team and reflexivity**		
*Personal Characteristics*		
1. Interviewer/facilitator	Which author/s conducted the interview or focus group?	Valeria Di Giuseppe,
		Daniela Popa, Raffaella Gualandi
2. Credentials	What were the researcher’s credentials? E.g. PhD, MD	PHD Student, RN, PHD
3. Occupation	What was their occupation at the time of the study?	Nurse and Nurse Director
4. Gender	Was the researcher male or female?	Females
5. Experience and training	What experience or training did the researcher have?	More than 10 years of experience, previous experience with qualitative research
*Relationship with participants*		
6. Relationship established	Was a relationship established prior to study commencement?	page 7
7. Participant knowledge of the interviewer	What did the participants know about the researcher? E.g., personal goals, reasons for doing the research	page 7–8
8. Interviewer characteristics	What characteristics were reported about the interviewer/facilitator? E.g., bias, assumptions, reasons and interests in the research topic	page 1
**Domain 2: study design**		
*Theoretical framework*		
9. Methodological orientation and Theory	What methodological orientation was stated to underpin the study? E.g., grounded theory, discourse analysis, ethnography, phenomenology, content analysis	page 8–9
*Participant selection*		
10. Sampling	How were participants selected? E.g., purposive, convenience, consecutive, snowball	page 7
11. Method of approach	How were participants approached? E.g., face-to-face, telephone, mail, email	page 7
12. Sample size	How many participants were in the study?	page 10
13. Non-participation	How many people refused to participate or dropped out? Reasons?	page 10
*Setting*		
14. Setting of data collection	Where was the data collected? E.g., home, clinic, workplace	page 7
15. Presence of non-participants	Was anyone else present besides the participants and researchers?	page 8
16. Description of sample	What are the important characteristics of the sample? E.g., demographic data, date	page 10
*Data collection*		
17. Interview guide	Were questions, prompts, guides provided by the authors? Was it pilot tested?	page 8
18. Repeat interviews	Were repeat interviews carried out? If yes, how many?	None
19. Audio/visual recording	Did the research use audio or visual recording to collect the data?	page 8
20. Field notes	Were field notes made during and/or after the interview or focus group?	page 8
21. Duration	What was the duration of the interviews or focus group?	page 8
22. Data saturation	Was data saturation discussed?	page 8
23. Transcripts returned	Were transcripts returned to participants for comment and/or correction?	None
**Domain 3: analysis and findings**		
*Data analysis*		
24. Number of data coders	How many data coders coded the data?	page 8–9
25. Description of the coding tree	Did authors provide a description of the coding tree?	page 8–9
26. Derivation of themes	Were themes identified in advance or derived from the data?	page 8–9
27. Software	What software, if applicable, was used to manage the data?	page 7
28. Participant checking	Did participants provide feedback on the findings?	Not applicable
*Reporting*		
29. Quotations presented	Were participant quotations presented to illustrate the themes/findings? Was each quotation identified? E.g., participant number	from page 10 to 16
30. Data and findings consistent	Was there consistency between the data presented and the findings?	from page 10 to 16
31. Clarity of major themes	Were major themes clearly presented in the findings?	from page 10 to 16
32. Clarity of minor themes	Is there a description of diverse cases or discussion of minor themes?	from page 10 to 16
